# ﻿*Vaccinium bidoupense* (Ericaceae), a new species from Vietnam and Laos, with notes on *V. petelotii* and *V. pseudobullatum*

**DOI:** 10.3897/phytokeys.266.168208

**Published:** 2025-11-07

**Authors:** Thi Thanh Huong Nguyen, Shuichiro Tagane, Leonid V. Averyanov, Van Son Dang, Peter W. Fritsch, Yi-Hua Tong, Phetlasy Souladeth, Maxim S. Nuraliev

**Affiliations:** 1 Institute of Biology, Vietnam Academy of Science and Technology, 18 Hoang Quoc Viet, Nghia Do Ward, Hanoi, 100000, Vietnam Institute of Biology, Vietnam Academy of Science and Technology Hanoi Vietnam; 2 Graduate University of Science and Technology, Vietnam Academy of Science and Technology, 18 Hoang Quoc Viet, Nghia Do Ward, Hanoi, 10072, Vietnam Graduate University of Science and Technology, Vietnam Academy of Science and Technology Hanoi Vietnam; 3 Kagoshima University Museum, Kagoshima University, Kagoshima, 890-0065, Japan Kagoshima University Kagoshima Japan; 4 Komarov Botanical Institute of the Russian Academy of Sciences, St. Petersburg 197376, Russia Komarov Botanical Institute of the Russian Academy of Science St. Petersburg Russia; 5 Institute of Life Sciences, Vietnam Academy of Science and Technology, Ho Chi Minh City, 700000, Vietnam Institute of Life Sciences, Vietnam Academy of Science and Technology Ho Chi Minh Vietnam; 6 Botanical Research Institute of Texas, 1700 University Drive, Fort Worth, Texas, 76107-3400, USA Botanical Research Institute of Texas Fort Worth United States of America; 7 State Key Laboratory of Plant Diversity and Specialty Crops & Laboratory of Plant Resources Conservation and Sustainable Utilization, South China Botanical Garden, Chinese Academy of Sciences, Guangzhou, Guangdong, 510650, China South China Botanical Garden, Chinese Academy of Sciences Guangzhou China; 8 South China National Botanical Garden, Chinese Academy of Sciences, Guangzhou, Guangdong, 510650, China South China National Botanical Garden, Chinese Academy of Sciences Guangzhou China; 9 Faculty of Forest Science, National University of Laos, Dongdok Campus, Xaythany District, Vientiane, Laos National University of Laos Vientiane Laos; 10 Department of Higher Plants, Biological Faculty, M.V. Lomonosov Moscow State University, 1, 12 Leninskie Gory, Moscow, 119234, Russia M.V. Lomonosov Moscow State University Moscow Russia; 11 Joint Russian-Vietnamese Tropical Scientific and Technological Center, 63 Nguyen Van Huyen Street, Cau Giay, Hanoi, 122000, Vietnam Joint Russian-Vietnamese Tropical Scientific and Technological Center Cau Giay Vietnam

**Keywords:** Central Highlands of Vietnam, Eastern Indochina, epiphytes, Tay Nguyen Plateau, Vaccinieae, *Vaccinium* sect. *Calcicolus*

## Abstract

*Vaccinium bidoupense*, a new species of Ericaceae from the Central Highlands of Vietnam and their vicinity in Laos, is described and illustrated. The new species is morphologically similar to *V. petelotii* and *V. pseudobullatum* in its relatively large leaf blades and umbel-like inflorescence but differs in having the following combination of characters: pale red to salmon pink corollas, and longer pedicels, calyx lobes, corolla tubes and stamen filaments. The distinctness of *V. bidoupense* is further supported by its allopatric distribution relative to both *V. petelotii* and *V. pseudobullatum*. The range of morphological variation of *V. petelotii* and *V. pseudobullatum* is clarified, and their analytical photographic images are published for the first time. Additionally, the distinction between indeterminate and determinate racemes in *Vaccinium* is revisited, and the taxonomic history of Vaccinium sect. Calcicolus is summarized.

## ﻿Introduction

*Vaccinium* L. is the largest genus of the tribe Vaccinieae in Ericaceae (Fang 1991; Fang and Stevens 2005; Vander Kloet and Dickinson 2009). The genus has a nearly cosmopolitan distribution and comprises about 450–500 species. Recent phylogenetic studies have demonstrated that *Vaccinium* and many of its traditionally defined sections are non-monophyletic (Kron et al. 2002; Becker et al. 2024). These findings, however, have not yet been reflected in the corresponding taxonomic rearrangements, and denser sampling is needed before a comprehensive reclassification of the tribe can be attempted (Becker et al. 2024). Pending updates of the formal classification based on phylogenetics, we here adopt the current circumscription and subdivision of *Vaccinium*.

In Eastern Indochina, 31 species of *Vaccinium* have been recorded, all of which are known from Vietnam. Of these, 20 species were listed by Nguyen and Ho (1996) and Nguyen (2003), whereas the illustrated guide by Ho (1999) comprised 21 species through the inclusion of *V. delavayi* Franch. Subsequently, 10 species of *Vaccinium* were added to the known flora of Vietnam (Vander Kloet and Paterson 2000; Nguyen et al. 2008; Hoang et al. 2016; Hummer et al. 2017; Averyanov et al. 2020; Vu-Huynh et al. 2020; Tong et al. 2022). Three species are known from Laos (Newman et al. 2007), and two species from Cambodia (Cho et al. 2016).

Here, we describe a species of *Vaccinium* new to science based on our original gatherings, as well as historical collections, made in the Central Highlands of Vietnam and in southern Laos. To facilitate the taxonomic assessment of the new species, we revise the morphological variation of its two similar species, the Asian *V. petelotii* Merr. and *V. pseudobullatum* W.P.Fang & Z.H.Pan and rectify the contradictions in the understanding of their floral structure. Finally, we discuss the circumscription of Vaccinium sect. Calcicolus Kloet, which was earlier proposed to include the species treated here.

## ﻿Materials and methods

Specimens of *Vaccinium* (or their images) kept in the following herbaria were examined: A, BRIT, DLU, FOF, FU, GXMG, GXMI, HITBC, HN, IBK, IBSC, K, KAG, KUN, LBG, LE, MO, MW, P, PE, SWFC, SZ, UC, US, VNM and WUK (herbarium acronyms follow Thiers, updated continuously). Small plant parts were observed with a hand magnifier. Terminology follows that of Fang and Stevens (2005), Beentje (2016) and Nuraliev et al. (2020). In each list of the examined specimens, the collections are arranged geographically (from NW to SE), and then chronologically. Distribution of the studied taxa in Vietnam is given in accordance with the official administrative division of the country as provided by the Vietnam Administrative Atlas (2015).

## ﻿Taxonomic treatment

### ﻿ Vaccinium petelotii

Taxon classificationPlantaeEricalesEricaceae

Merr., Univ. Calif. Publ. Bot. 13(6): 138 (1926).

5F42E032-4870-55BF-BAC8-728F76B5FA96

Fig. 1

= Agapetes parviflora Dunn, J. Linn. Soc., Bot. 35: 515 (1903). Type: China. Yunnan Province • Meng-tze [Mengzi County], *A. Henry 10488A* [fr.] (lectotype, designated by Sleumer 1941: 446, US, 00116973, image!).

#### Type.

Vietnam. Lao Cai Province • Chapa [Sapa], 1500 m a.s.l., April 1925, *P.A. Pételot 1772* [young fr.] (holotype: UC, UC259692, image!; isotypes: A, 00015962, image!; P, P00647843, image!; US, 00116935, image!).

#### Notes.

Based on the specimens cited in Appendix 1, we have clarified the morphological variation of the species as follows:

1. In the treatments by Fang (1991) and Fang and Stevens (2005), *Vaccinium petelotii* is described as having corollas 6–11 mm long. However, the specimens of this species examined in our study bear corollas 4–5 mm long, consistent with the description by, e.g., Huang and Fang (1991) who indicated corollas about 6 mm long. At the same time, the specimen *P.I Mao 82-172* (SWFC, 00004475 & 00004478) from Yunnan assigned to *V. petelotii* by R.C. Fang in 1984 demonstrates remarkably larger flowers with corollas up to 12 mm long. We suppose that the indications of large corollas were based solely on this specimen. Apart from flower size, this specimen differs from typical *V. petelotii* in larger leaves and shorter pedicels that remain straight (rather than becoming curved) when fruiting. We therefore exclude this specimen from *V. petelotii* and consider it a possible hybrid between *V. petelotii* and *V. pseudobullatum*.
2. Fang and Stevens (2005) indicated the stamen filaments of *Vaccinium petelotii* as being 0.5 mm long, in which case the anthers would seem to be nearly sessile. We could not detect any reason for this indication and consider it erroneous because the specimens of this species studied here uniformly have filaments 2.5–3 mm long.
3. The ratio of anther tubules to thecae was indicated by Fang and Stevens (2005) as ca. 3 for *Vaccinium petelotii*, which contradicts our observations (1.6–2.0) as well as the treatments by Fang (1991) and Huang and Fang (1991), both of which state a ratio of 1.5.

#### Distribution.

S China (SE Yunnan, SW Guangxi), N Vietnam (Lai Chau, Lao Cai).

**Figure 1.**
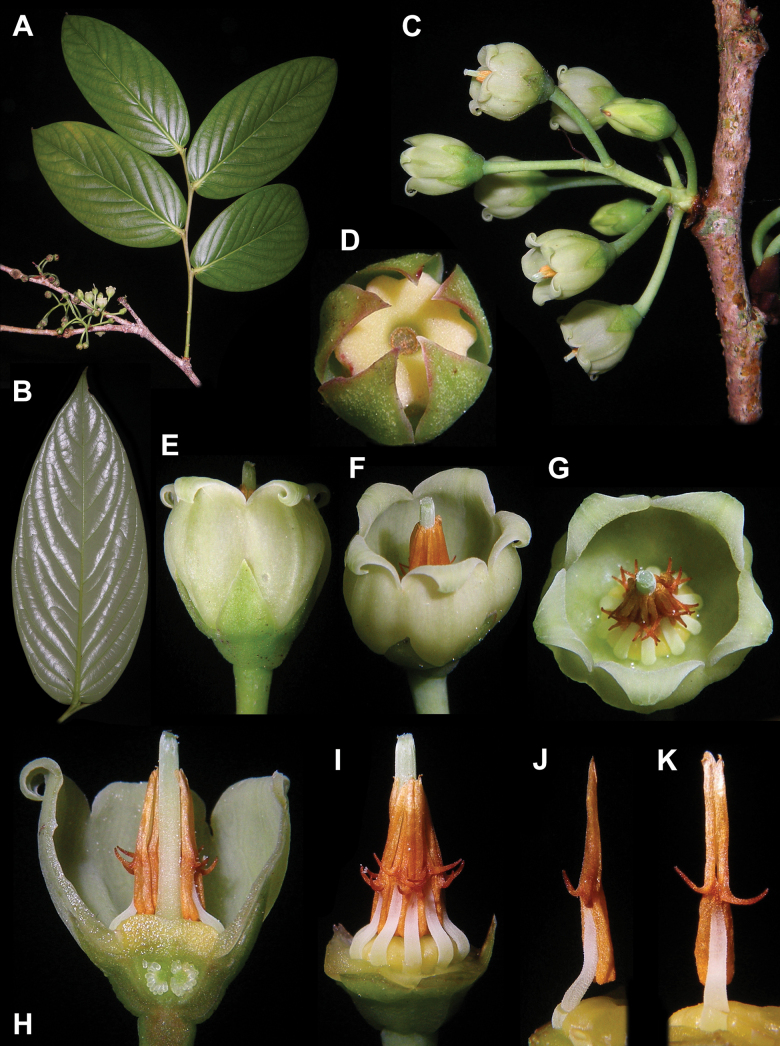
*Vaccinium petelotii*. A. Branch with leaves and flowers; B. Leaf blade, abaxial view; C. Inflorescence, lateral view; D. Post-anthetic calyx and disk; E–G. Flower, lateral, oblique and apical view; H. Longitudinal section of flower; I. Flower with perianth removed to show androecium and pistil; J. Stamen, lateral view; K. Stamen, dorsal view. Photos by M.S. Nuraliev, based on *M.S. Nuraliev 2359*.

### ﻿ Vaccinium pseudobullatum

Taxon classificationPlantaeEricalesEricaceae

W.P.Fang & Z.H.Pan, Acta Phytotax. Sin. 19(1): 108 (1981).

6103E0F1-F5D7-58A7-BF59-3E10CA25EDD5

Fig. 2

#### Type.

China. Yunnan Province • Malipo County, Tiechang, 1200 m a.s.l., 20 February 1940, *C.W. Wang 86988* [fl.] (holotype: PE; isotypes: IBK, IBK00302352!; IBSC, 0457816!; KUN, 12088909! & 12088910!; SZ, 00126560!; WUK, 0268189, image!).

#### Notes.

Based on the specimens cited in Appendix 1, we have clarified two androecial features of the species:

1. The length of the stamen filaments was indicated in the protologue (Fang and Pan 1981) as 1–1.5 mm, which was repeated in Fang and Stevens (2005). Fang (1991) and Huang and Fang (1991) considered the range as 1–2 mm. In most of the specimens studied here, however, the filaments are 3–4 mm long. Both flowering gatherings listed by Fang and Pan (1981; *Wang 86988* and *Feng 13490*) bear flower buds (and no fully open flowers), and we suppose it to be the reason that they indicate shorter filaments than those observed by us. Their description was likely reproduced in the subsequent account without additional investigation of flower structure.
2. The ratio of anther tubules to thecae was uniformly indicated as 2–3 for this species (Fang 1991; Huang and Fang 1991, including fig. 105 (9, 10); Fang and Stevens 2005), whereas we observed it to be mostly 1.1–1.3. We therefore treat this feature as more variable than earlier thought.

#### Distribution.

S China (SE Yunnan, SW Guangxi), N Vietnam (Cao Bang, Ha Giang). Until recently, the species was considered to be endemic to Yunnan; it was reported from Guangxi by Wei (2023) and from Vietnam by Averyanov et al. (2020).

**Figure 2.**
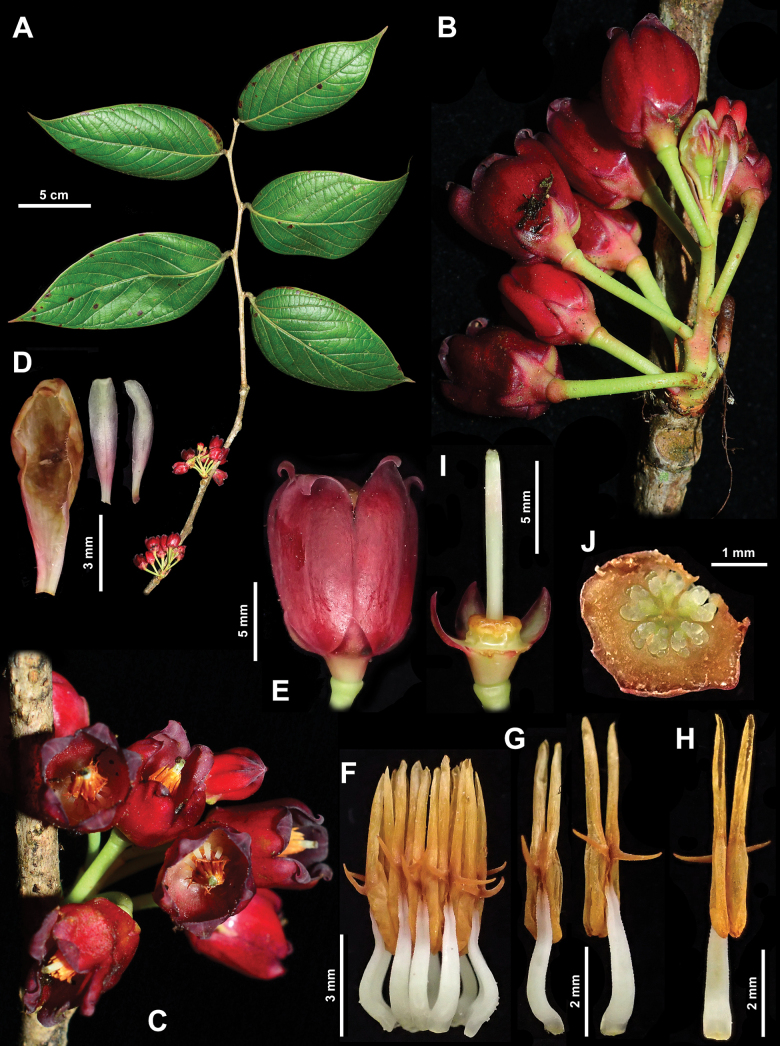
*Vaccinium pseudobullatum*. A. Branch with leaves and flowers; B. Inflorescence, lateral view; C. Inflorescence, with front view of flowers; D. Bract and two bracteoles; E. Flower, lateral view; F. Androecium; G. Antepetalous (left) and antesepalous (right) stamens, dorsal view; H. Stamen, ventral view I. Three calyx lobes, ovary, disk and style; J. Cross section of ovary. Photos by Y.H. Tong, based on *B.M. Wang TYH-965A*.

### ﻿ Vaccinium bidoupense

Taxon classificationPlantaeEricalesEricaceae

Smitinand ex Y.H.Tong, N.T.T.Huong & Tagane sp. nov.

FB85C397-5D32-5E61-BC6F-9A9F2F59C461

urn:lsid:ipni.org:names:77371640-1

Figs 3, 4, 5, 6

#### Type.

Vietnam. Kon Tum Province • NW slopes of Ngoc Linh Mountain system at 1800–1900 m a.s.l., open places in primary evergreen dense mountain forest, 23 February 1995, *L.V. Averyanov, N.T. Ban, N.Q. Binh, A. Budantzev, L. Budantzev, N.T. Hiep, D.D. Huyen, P.K. Loc, N.X. Tam, G. Yakovlev VH053* [fl.] (holotype: HN!; isotypes: BRIT, BRIT402341!; LE, LE01041972!; MO, 5168102!; P, P04485557! & P04483084!).

#### Diagnosis.

*Vaccinium bidoupense* is morphologically similar to *V. petelotii* and *V. pseudobullatum* in its relatively large leaf blades and umbel-like inflorescence with short rachis. It differs from *V. petelotii* in having longer calyx lobes (5–6 mm vs. 2.5–3 mm), pale red to salmon pink (vs. pale green) corolla, a longer corolla tube (11–13.5 mm vs. 3.5–4.5 mm), and longer stamens (14–16 mm vs. 5–6.5 mm). The new species differs from *V. pseudobullatum* in having leaf blades with somewhat less obvious tertiary veins, and longer pedicels (7–14 mm vs. 4–7 mm), corolla tube (11–13.5 mm vs. 8–9 mm) and stamen filaments (8–9 mm vs. 1–4 mm) (Table 1).

**Figure 3.**
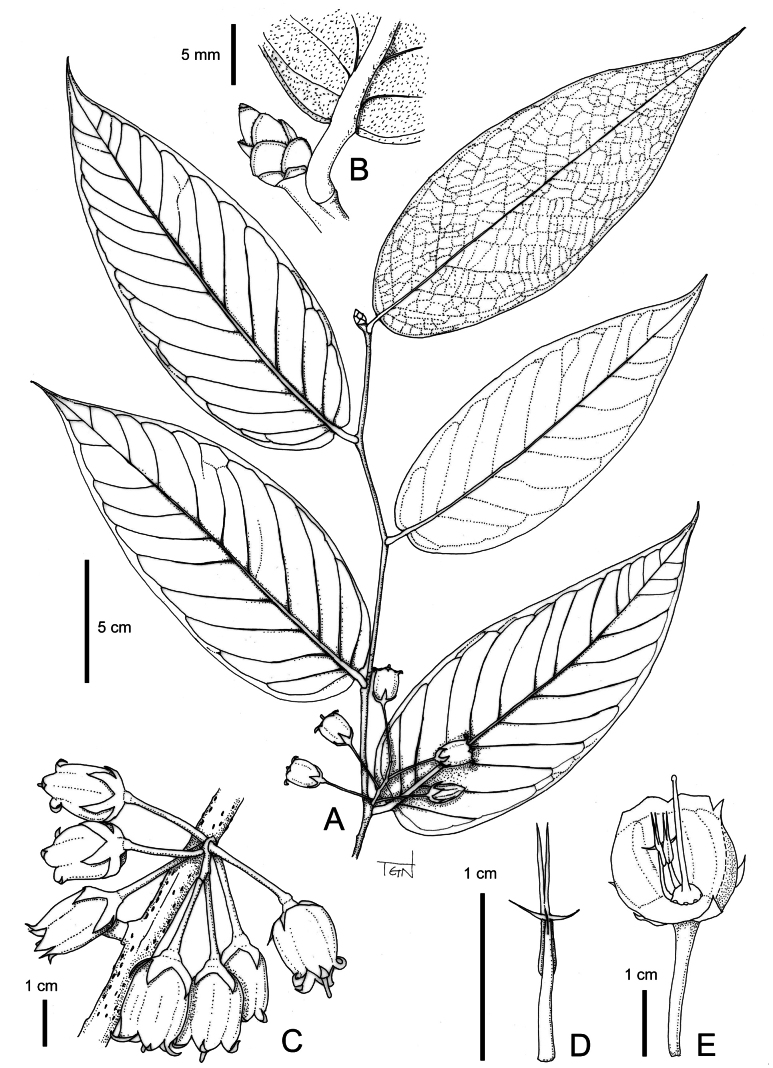
*Vaccinium bidoupense*. A. Flowering branch; B. Bud and base of leaf (abaxial side) showing dense appressed trichomes; C. Inflorescence; D. Stamen, dorsal view; E. Flower with part of corolla and some stamens removed to show disk and style. Drawn by S. Tagane.

**Table 1.** A comparison of *Vaccinium bidoupense*, *V. petelotii* and *V. pseudobullatum.* The data on the latter two species were obtained from the cited literature sources and the examined specimens listed in Appendix 1.

Characters	V. bidoupense	V. petelotii	V. pseudobullatum
References	this study	Dunn (1903), Merrill (1926), Dop and Trochain-Marquès (1931), Fang (1991), Huang and Fang (1991), Fang and Stevens (2005)	Fang and Pan (1981), Fang (1991), Huang and Fang (1991), Fang and Stevens (2005)
Leaf blade: shape	ovate-elliptic to lanceolate	oblong or narrowly ovate	elliptic to ovate
Leaf blade: tertiary veins	prominent in submarginal area, stout	prominent throughout, slender	prominent throughout, stout
Petiole: length (mm)	3–12	3–6	6–8
Pedicel: length (mm)	7–14	9–15	4–7
Calyx: lobe length (mm)	5–6	2.5–3	4–6
Corolla: color	pale red to salmon pink	pale green (usually described as cream-colored)	red, pink or white
Corolla: length of tube (mm)	11–13.5	3.5–4.5	8–9
Stamen: length (mm)	14–16	5–6.5	6.5–7
Stamen: filament length (mm)	8–9	2.5–3	(1–)2–3(–4)
Stamen: length ratio of anther tubules to thecae	ca. 1.3	1.5–2	1.1–1.3(–3)
Fruiting pedicel	straight	curved	curved
Distribution	Laos (Sekong), S Vietnam (Kon Tum, Lam Dong, Quang Nam)	S China (SE Yunnan), N Vietnam (Lai Chau, Lao Cai)	S China (SE Yunnan, SW Guangxi), N Vietnam (Cao Bang, Ha Giang)

#### Description.

***Shrubs***, epiphytic or epilithic, evergreen; shoots pendent, ca. 2–5(–10) m long, sometimes rooting at nodes. Branches more or less angled, glabrous, densely lenticellate. ***Leaves*** non-distichously alternate (but leaves of a branch often arranged in one plane due to twisted petioles), evenly scattered. ***Petiole*** 0.3–1.2 cm long, glabrous. ***Leaf blade*** ovate to lanceolate or sometimes elliptic, 12–22 × 4.7–11.7 cm, 2.0–3.7 times as long as wide, coriaceous; adaxial surface green, glabrous; abaxial surface pale green, densely covered with appressed dark brown trichomes (trichomes caducous, leaving faintly punctate scars); base rounded, subtruncate or shallowly cordate, rarely cuneate or prominently cordate; margin entire, slightly revolute, with 1 basal gland per side, glands 1–1.5 mm in diam.; apex acute to acuminate or shortly caudate; median vein and secondary veins impressed adaxially, strongly raised abaxially; secondary veins pinnately arranged, 10–14 on each side of median vein, usually meeting in a closed loop and forming an intramarginal vein; tertiary veins more prominent in submarginal area than in the rest of leaf blade, stout, reticulate, slightly impressed or sometimes flat adaxially, slightly raised abaxially. ***Inflorescences*** axillary, 1 per axil, on leafy shoots and older leafless branches, sessile (peduncle absent), racemose, umbel-like, determinate (developing mostly within the confines of the perennating buds), 4–10-flowered; rachis pale green, 0.5–2 cm long, glabrous; bracts caducous, crimson, ovate, 1.0–1.5 × 0.5–0.6 cm, thinly coriaceous, glabrous, margin entire, apex obtuse. ***Flowers*** pendent, articulated with pedicels, 5-merous. ***Pedicels*** red to pale green, 0.7–1.4 cm long, glabrous; bracteoles 2, attached at base of pedicel, subopposite, acicular, early caducous. ***Calyx*** (excluding ovary) pale red to salmon pink, becoming greenish with age, at base appressed to corolla and distally diverging to spreading, glabrous on both sides; tube ca. 1 mm long; lobes 5, triangular-lanceolate, 5–6 × 2–3 mm, distinctly 1-veined. ***Corolla*** pale red to salmon pink, glabrous outside and inside; tube cupulate, 11–13.5 × 13–14 mm; lobes 5, strongly recurved, triangular to broadly triangular, 3–4 × 4–5 mm. ***Stamens*** 10, dimorphic with respect to spurs, included in corolla, free from each other, 14–16 mm long; filaments white to pale pink, slightly incurved, 8–9 mm long, glabrous in proximal half and puberulent in distal half; anthers tightly appressed to style, golden yellow, dorsifixed, 7–9 mm long, each with 2 spurs; thecae 3–3.5 mm long, slightly papillate; tubules parallel, 4–6 mm long (ca. 1.3 times as long as thecae), opening by oblique ventral apical pores; spurs borne dorsally at base of tubules, strongly curved, ca. 1 mm long, those on antesepalous stamens extending laterally outside of antepetalous anthers and overlapping with spurs of next antesepalous stamens, those on antepetalous stamens strongly hooked outward below spurs of antesepalous stamens. ***Ovary*** inferior, pale red to salmon pink, obconic, terete, 1–2 × 2–2.5 mm, glabrous, pseudo-10-locular; disk pale yellow, annular, broadly dome-shaped, ca. 2.5 mm diam., glabrous; style 1.4–1.6 cm long, exserted from corolla for 2.5–4 mm, linear, glabrous; stigma truncate. ***Infructescences*** with rachis 1–3 cm long. ***Fruiting pedicel*** 1.2–2.4 cm long. ***Fruit*** (when young) globose, ca. 6 mm in diam., glabrous, with persistent calyx lobes 5–6 mm long. ***Mature fruits and seeds*** unknown.

**Figure 4.**
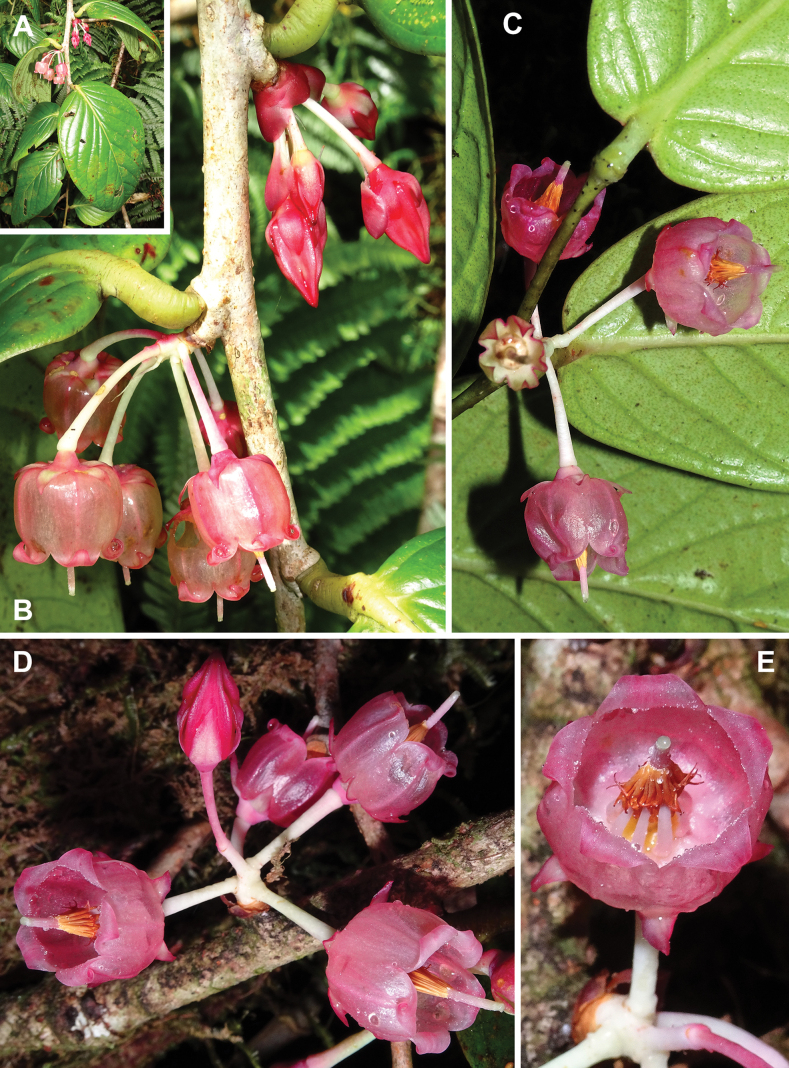
*Vaccinium bidoupense*. A. Flowering branch; B–D. Inflorescences; E. Flower, apical view. Photos by L.V. Averyanov, based on *Averyanov et al. AL2537*.

#### Ecology and phenology.

*Vaccinium bidoupense* grows as an epiphyte on tree trunks or as a lithophyte on mossy rocks in broad-leaved and mixed forests at elevations of 1100–2200 m. It flowers from October to February; young fruits are documented from May to June.

#### Distribution.

*Vaccinium bidoupense* is known from Laos (Sekong Province) and Vietnam (provinces Kon Tum, Lam Dong, and Quang Nam). The species is therefore endemic to the area comprising the Central Highlands of Vietnam (also called Tay Nguyen Plateau) and their extension in Laos known as Dak Cheung (or Dakchung) Plateau, being broadly distributed across this region. The region comprises three main mountainous areas, i.e., Dak Lak Plateau, Kon Tum—Gia Lai Plateau and Langbian Plateau (Poyarkov et al. 2021), and *V. bidoupense* is documented to inhabit the latter two areas. As the known locations suggest, the species probably occurs throughout the Central Highlands, and its presence in the provinces Dak Lak, Dak Nong and Gia Lai is especially probable.

**Figure 5.**
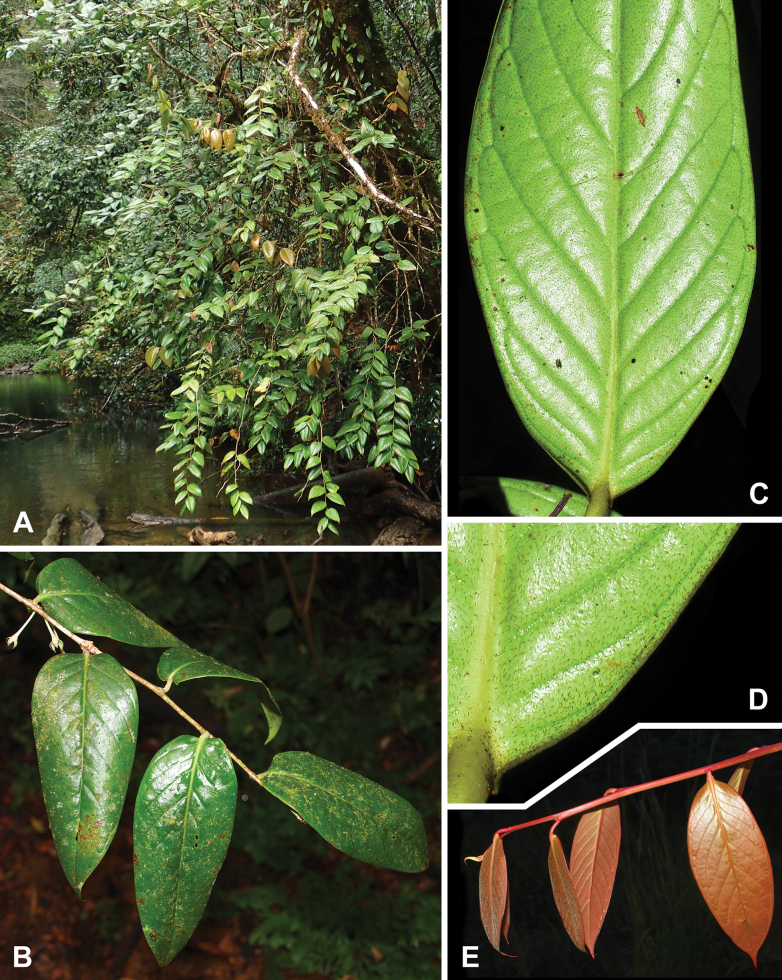
*Vaccinium bidoupense*. A. Habit; B. Leafy branch; C. Leaf blade, abaxial view; D. Base of leaf, abaxial view, showing appressed dark brown trichomes; E. Young leafy branch. Photos by S. Tagane, A. unvouchered; B. based on *Toyama et al. V1857*; C–E. based on *Tagane et al. V4287*.

#### Etymology and history of documentation of *Vaccinium bidoupense*.

The species name “*Vaccinium bidoupense*” was initially proposed by Tem Smitinand on the determination slips of three of Poilane’s specimens kept in P (*30292*, *30763* and *30909*, included below as paratypes of the species). Two of these specimens originated from the Bidoup Massif (and *30292* was collected nearby), which is reflected in the species epithet. Although such a name has never been formally published until now, it was used by Vander Kloet and Avery (2007) (as “*V. bidoupense* Smithin”) who included the specimen *Poilane 30763* in a morphophenetic analysis of stamen characters in the tribe Vaccinieae. Here, we follow Smitinand’s choice of epithet to ensure the clearness of the species identity and to acknowledge his recognition of this distinct species.

**Figure 6.**
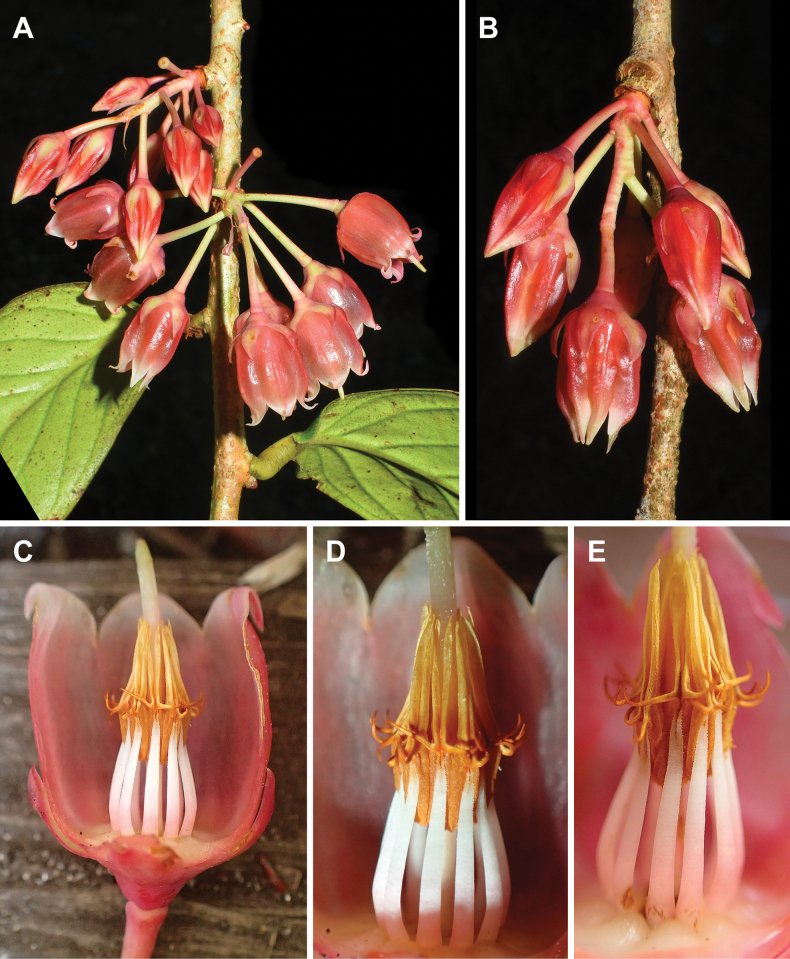
*Vaccinium bidoupense*. A, B. Inflorescences; C. Flower with a half of perianth removed; D, E. Androecium. Photos by S. Tagane, based on *Tagane et al. V4287*.

#### Conservation status.

*Vaccinium bidoupense* is an integral element of highland vegetation of the Central Highlands of Vietnam (including their extension in Laos) with an expected total extent of occurrence (EOO) of about 55,000 km^2^, which does not fit any of the threatened IUCN Red List conservation categories (IUCN, Standards and Petitions Committee 2025). In intact habitats of montane forests, the species meets no damage factors. However, the vast degradation of primary vegetation across the distribution area of the species, including the legally protected territories, leads to a continuing decline in the extent of its occurrence, quality of habitat, number of populations, and number of mature individuals. In this connection, we preliminarily assess *V. bidoupense* as Near Threatened (NT); we also consider that the species is close to being qualified as Vulnerable (VU) in the near future.

#### Additional specimens examined (paratypes).

**Laos. Sekong Province** • [Dak Cheung District,] new road along the border with Vietnam, 15°34′36.0′′N, 107°20′11.5′′E, 1224 m a.s.l., 17 July 2021, *P. Souladeth, M. Soukhavong, N. Thongphakdee, T. Boutavong 1141* [st.] (FOF, FOF0009576; KAG, KAG181621). **Vietnam. Quang Nam Province** • Nam Giang District, Song Thanh Nature Reserve, 15°33′07′′N, 107°23′02′′E, 1100 m a.s.l., 6 May 2019, *M.S. Nuraliev 2509* [young fr.] (BRIT, BRIT1150627; IBSC; MW, MW0758862, MW0758863 & MW0758864). **Lam Dong Province** • N of Dalat, 1500 m a.s.l., 2 September 1940, *E. Poilane 30292* [young fr.] (L, L.3786302, image; P, P04484682, P04484683, P04484684, P04484685, P05244320 & P05244322, images) • Bidoup Massif, 2200 m a.s.l., 12 October 1940, *E. Poilane 30763* [fl.] (L, L.3786303, image; P, P00647856, P04484686, P04484688, P05244702 & P05244703, images) • ibid., 2000 m a.s.l., 14 October 1940, *E. Poilane 30909* [fl.] (P, P04484687, P05244317 & P05244321) • Lac Duong District, Da Chay Municipality, 29 km to NE from Dalat City, 12°6′N, 108°39′E, 1900–2000 m a.s.l., 23 March 1997, *L.V. Averyanov, N.Q. Binh, P.K. Loc VH3118* [st.] (HN; LE, LE01041973; MO, MO-5172316) • ibid., 12°6′N, 108°39′E, 2150 m a.s.l., 1 May 1997, *L.V. Averyanov, N.Q. Binh, N.T. Hiep, P.K. Loc, P. Lowry VH4463* [fr.] (HN; LE, LE01041976; MO, MO2080998; MW, MW1045967; P, P04483084) • Lac Duong District, Bidoup Nui Ba National Park, 12 October 2011, *N.H. Xia, J.B. Ni, Y.H. Tong & X.R. Zheng TYH-1070* [st.] (IBSC) • ibid., SE of Giang Ly station, 12°11′07.7′′N, 108°41′14.6′′E, 1509 m a.s.l., 23 December 2012, *A.N. Kuznetsov, S.P. Kuznetsova, A.N. Demidova, N.G. Prilepsky 404* [st.] (MW, MW0750311) • ibid., 12°10′21.03′′N, 108°41′49.76′′E, 1504 m a.s.l., 19 November 2014, *H. Toyama, S. Tagane, V.S. Dang, H. Nagamasu, A. Naiki, H. Tran, C.J. Yang et al. V1857* [fl.] (FU; VNM) • ibid., 12°11′11.18′′N, 108°42′53.12′′E, 1639 m a.s.l., 23 February 2016, *S. Tagane et al. V4287* [fl.] (DLU; FU; VNM) • ibid., 12°11′09.23′′N, 108°42′55.46′′E, 1637 m a.s.l., 22 December 2018, *S. Tagane et al. V9614* [fl.] (DLU; FU; KAG, KAG127364) • ibid., 22 April 2019, *T. Yahara et al. V9879* [young fr.] (DLU; FU; KAG, KAG182510) • ibid., 12°4.076′N, 108°38.927′E, 2000–2200 m a.s.l., 8 November 2023, *L.V. Averyanov, V.C. Nguyen, B.V. Truong, T. Maisak AL2537* [fl.] (LE, LE01253935, photos LE 01124590).

## ﻿Discussion

### ﻿Notes on inflorescence diversity in *Vaccinium*

The entire genus *Vaccinium* is characterized by open racemose inflorescences except for the species with single axillary flowers (e.g. Stevens et al. 2004; Fang and Stevens 2005). At the same time, the inflorescences vary in the timing and pattern of their development. As highlighted by Vander Kloet and Dickinson (2005), species of the genus fall into two rather distinct groups differing in having indeterminate and determinate racemes. Vander Kloet and Dickinson (2005) defined the two types as follows: in indeterminate inflorescences, the rachis (termed “floral axis” by these authors) develops after the opening of the inflorescence bud, and in determinate inflorescences it develops within the inflorescence bud. This distinction is consistent with the ideas of Claßen-Bockhoff and Bull-Hereñu (2013; see also Claßen-Bockhoff et al. 2021) who considered “inflorescences sensu stricto” (with indeterminate apical meristem, although usually limited in its activity) and “floral units” (with determinate apical meristem that lacks stem cells). We suggest that a feature allowing a stricter discrimination of the two types of racemes and at the same time offering their easier recognition is the emergence of new flowers during the growth of the raceme beyond the confines of the inflorescence bud. In indeterminate racemes, the apical meristem continues to produce new flowers well after the bud opening, which often leads to a long duration of the raceme flowering and to markedly differing phenology of individual flowers along the rachis (including even the co-existence of young fruits at the base of the inflorescence with underdeveloped flower buds at its apex). Conversely, in determinate racemes all the flowers are formed by the time of bud opening. In this case, although the flower buds are sometimes pronouncedly unequal in size at the stage of opening of the inflorescence bud, the flowers function more or less synchronously.

An additional difference between the types of racemes (already highlighted by Vander Kloet and Dickinson 2005) is the tendency of the bracts subtending each pedicel to be leaf-like or scale-like and often small in indeterminate racemes, whereas the determinate racemes usually have elaborate and large bracts that act as scales of showy inflorescence buds. It should be noted that the features that define the two types are not related to the number of flowers per raceme, as this varies greatly within each type.

Although the distinctness of the two types of racemes was confirmed for the species of *Vaccinium* closely examined so far (e.g., by Vander Kloet and Dickinson 2005, 2009; Nuraliev et al. 2020; Ye et al. 2021; Tong et al. 2022), it is still to be tested on a broader sampling, and an assessment is needed as to the extent of correspondence with the sections of *Vaccinium* and phylogeny of the tribe Vaccinieae. For the moment, and based on the clarification provided above, we here reconsider the racemes of *V. glaucorubrum* (C.Y.Wu) Y.H.Tong & P.W.Fritsch described as indeterminate in our earlier study (Tong et al. 2022) as being determinate. In particular, all the flowers are formed (and readily visible) in this species by the time of the opening of the inflorescence bud and are subtended by brightly colored rhomboid bracts ca. as long as the flowers.

Vander Kloet and Dickinson (2005) recognized three groups of species within the tribe Vaccinieae based on their morphophenetic analysis of inflorescence characters. These authors stated that the groups (designated as A, B and C) are largely in agreement with the primary subdivisions (III, II and I, correspondingly) in the polytomic key to the sections of *Vaccinium* compiled by Sleumer (1941, p. 413). However, the morphological characterizations provided by Vander Kloet and Dickinson (2005) for their groups differ considerably from Sleumer’s couplets; in particular, Sleumer did not mention the indeterminate vs. determinate racemes. Most importantly, we uncovered about a dozen sections of *Vaccinium* whose placement represent discrepancies between Vander Kloet and Dickinson’s groups and Sleumer’s key.

### ﻿Taxonomic distinctness of *Vaccinium bidoupense*

*Vaccinium bidoupense* differs from most of its congeners in having the following combination of morphological traits: non-tuberous stem bases or roots, leaf blades with entire margins and without glandular setae on the median vein abaxially, and determinate racemes that are umbel-like from the pedicels being approximately as long as the rachis. It shares this set of features with only two species of the genus, viz., *V. petelotii* and *V. pseudobullatum*. Comparison of the three species is provided in the diagnosis. It is remarkable that the close similarity of the new species with *V. petelotii* was also assumed by Pham Hoang Ho, who identified the P specimens *in sched.* as “Vaccinium petelotii Merr. var. bidoupense Smitin. ex Pham-Hoang” (this varietal name, however, has never appeared in any publications).

Several collections assigned here to *Vaccinium bidoupense* (*Averyanov et al. VH053*, *VH3118*, *VH4463*, and *Poilane 30909*) were earlier identified *in sched.* as V. dunalianum var. megaphyllum Sleumer. However, *V. bidoupense*, and in particular the specimens in question, readily differs from the entirety of *V. dunalianum* Wight (Fang and Stevens 2005) in having umbel-like racemes with the rachis not longer than 2 cm and pedicels 0.7–1.4 cm long (vs. elongate racemes with rachis 3–7 cm long and pedicels 0.5–0.8 cm long) and much longer corolla tubes (11–13.5 mm vs. 3.5–4.5 mm long).

### ﻿History of Vaccinium sect. Calcicolus, with special reference to *V. bidoupense*

When establishing Vaccinium sect. Calcicolus, Vander Kloet and Dickinson (2005) placed eight species in the section, including *V. dunalianum* and *V. petelotii* discussed above. Shortly thereafter, Vander Kloet and Avery (2007) listed “*V. bidoupense*” as belonging to this section. Vander Kloet and Dickinson (2005, 2009) stated that the section is characterized by an evergreen habit, dimorphic perennating buds (the inflorescence buds at least three times as large as the vegetative buds), a determinate inflorescence (developing entirely within the confines of an enlarging perennating bud), large caducous inflorescence bracts, a pseudo-10-locular ovary, 2–5 seeds per locule, and soft seed testa (see also discussion of the section by Nuraliev et al. 2020). *Vaccinium pseudobullatum* (considered above as one of the two species most similar to *V. bidoupense*) was not mentioned by Vander Kloet and Dickinson (2005, 2009), nor by Vander Kloet and Avery (2007), but is consistent with Vaccinium sect. Calcicolus morphologically.

Vander Kloet and Dickinson (2009) emphasized the morphological similarity of *Vaccinium lanigerum* Sleumer to certain species of Vaccinium sect. Calcicolus. Consequently, they united Vaccinium sect. Calcicolus with Vaccinium sect. Pseudocephalanthos C.Y.Wu & R.C.Fang (but erroneously chose the former name for the expanded section, although the latter name has a nomenclatural priority). *Vaccinium lanigerum*, however, is characterized by swollen stem bases or roots (Y.H. Tong, personal observation in natural habitat), markedly differing in this respect from Vaccinium sect. Calcicolus (see Nuraliev et al. 2020). For this reason, we do not here follow the merger proposed by Vander Kloet and Dickinson (2009).

More recently, Ye et al. (2021) transferred *Vaccinium glaucophyllum* C.Y.Wu & R.C.Fang to Vaccinium sect. Calcicolus based on its overall morphology, and Tong et al. (2022) segregated *V. glaucorubrum* from *V. gaultheriifolium* (Griff.) Hook.f. ex C.B.Clarke, the type species of Vaccinium sect. Calcicolus. Finally, Tong et al. (2021) and Guo et al. (2023) placed their newly described *V. motuoense* Y.H.Tong & Y.J.Guo and *V. usneoides* Y.H.Tong, Y.J.Guo & Ting Zhang in the section. Altogether, 14 species have been explicitly placed in Vaccinium sect. Calcicolus. The morphological basis for these placements, however, largely remains questionable, and at the same time more than a dozen Asian species of *Vaccinium* possess the set of morphological characters outlined above but have not been analyzed with respect to their possible inclusion in Vaccinium sect. Calcicolus. Given all the uncertainties outlined above, we here refrain from assigning *V. bidoupense* to either Vaccinium sect. Calcicolus or any other formal infrageneric subdivision.

Becker et al. (2024) demonstrated polyphyly of Vaccinium sect. Calcicolus (*sensu* Vander Kloet et al.): of the four species sampled in their phylogenetic study, the clade *V. gaultheriifolium* + *V. glaucoalbum* Hook.f. ex C.B.Clarke is sister to Vaccinium sect. Aethopus Airy Shaw, and the clade *V. chunii* Merr. ex Sleumer + *V. dunalianum* groups with the species of Vaccinium sect. Conchophyllum Sleumer and Vaccinium sect. Galeopetalum J.J.Sm. (but Becker et al. 2024 erroneously placed *V. glaucoalbum* into Vaccinium sect. Galeopetalum at least as based on Vander Kloet and Dickinson 2005). Because no phylogenetic data are available for *V. bidoupense*, *V. petelotii* or *V. pseudobullatum*, it is currently not possible to establish their relationships. Phylogenetic reconstruction with expanded species sampling is strongly needed for comprehensive reassessment of Vaccinium sect. Calcicolus and the other sections of Asian tropical *Vaccinium*.

## Supplementary Material

XML Treatment for Vaccinium petelotii

XML Treatment for Vaccinium pseudobullatum

XML Treatment for Vaccinium bidoupense

## ﻿Additional information

### Use of AI

AI was not used in the preparation of the manuscript.

### Author ORCIDs

Shuichiro Tagane https://orcid.org/0000-0002-1974-7329

Leonid V. Averyanov https://orcid.org/0000-0001-8031-2925

Peter W. Fritsch https://orcid.org/0000-0002-3606-663X

Yi-Hua Tong https://orcid.org/0000-0002-5034-005X

Phetlasy Souladeth https://orcid.org/0000-0001-8563-1773

Maxim S. Nuraliev https://orcid.org/0000-0001-8291-2633

### Data availability

All of the data that support the findings of this study are available in the main text.

## ﻿Appendix 1

**List of examined herbarium specimens of *Vaccinium petelotii* Merr. and *V. pseudobullatum* W.P.Fang & Z.H.Pan.**

***Vaccinium petelotii*. China. Yunnan Province** • Lüchun County, Huanglianshan Mountain, Hydrologic Station, 1700–1900 m a.s.l., 16 October 1995, *S.K. Wu, Y.M. Shui, Y.P. Yang, L.H. Liu, J.H. He, J. Murata, H. Nagamasu, T. Sugawara, X. Chen & N. Murakmi 435* [fl. bud] (PE, 02055207) • ibid., 1600 m a.s.l., 21 December 2000, *L.M. Zhou 017* [young fr.] (KUN, 1254591) • ibid., 1600–1700 m a.s.l., 20 February 2006, *Y.M. Shui, W.H. Chen, et al. 70086* [young fr.] (PE, 02054616) • ibid., 1600–1700 m a.s.l., 22 February 2006, *Y.M. Shui, W.H. Chen, et al. 70128* [young fr.] (PE, 02054620) & *70139* [young fr.] (PE, 02054630 & 02054612) • ibid., 22°53′21′′N, 102°17′45′′E, 1771 m a.s.l., 15 March 2012, *Y.J. Guo, X.Z. Nie, Q.Y. Huang, J.H. He, L.H. Bai 12CS4832* [young fr.] (KUN, 1019832) • ibid., along the XG94 road from the Huanglianshan Mountain pass to Lüchun County, 22°55′54.34′′N, 102°16′56.87′′E,1845 m a.s.l., 22 December 2014, *T. Zhang, C. Liu, Z.M. Zhu 14CS6694* [fr.] (KUN, 1446600) • Jinping County, near the Sino-Vietnam border, 1700 m a.s.l., 1 April 1958, *Q. Huang 335* [fl.] (PE, 00438406 & 02054614) • Jinping County, Kui-he Nature Reserve Station, 1700 m, 8 October 1996, *S.K. Wu, L.H. Liu, Y.M. Shui, Y.P. Yang, J. Murata & S. Akiyama 4043* [fl.] (PE, 02054610) • Jinping County, Shuiduicun, Fenshuiling, 1809 m a.s.l., 21 October 1996, *S.K. Wu, L.H. Liu, Y.M. Shui, Y.P. Yang, J. Murata & S. Akiyama 4447* [fl.] (PE, 02055210) • ibid., 21 May 1956, *Sino-Soviet Expedition 3043* [fl.] (PE, 02054631) • Jinping County, Yongping Xiang, Hetouzhai, 1900 m a.s.l., 12 May 1956, *Sino-Soviet Expedition 1371* [fr.] (IBSC, 0456966; PE, 02054613) • Meng-tze [Mengzi County], *A. Henry 10488A* [fr.] (lectotype of *Agapetes parviflora* Dunn; US, 00116973, image) • Meng-tze [Mengzi County], *A. Henry 10488* (remaining syntype of *Agapetes parviflora* Dunn; A, 00121932, not seen) • Meng-tze [Mengzi County], 1901, *A. Henry 13306* [fl.] (A, 0121926, not seen; K, K000780614, image) • Pingbian County, 7 March 1934, *Y. Tsiang 13341* [fr.] (IBSC, 0456963 & 0456964) • Pingbian County, Mi-mi, 1500 m a.s.l., 13 November 1939, C.W. Wang 82765 [fr.] (KUN, 0234170; SZ, 00125781) • Pingbian County, Daweishan, Adakou, Niuchang, 1500 m a.s.l., 16 June 1956, *Sino-Soviet Expedition 3569* [young fr.] (IBSC, 0456967; PE, 02054629) • Pingbian County, Daweishan Reservoir, 22°54′N, 103°40′E, 2 November 1990, *Anonymous 22041* [young fr.] (HITBC 100568) • Pingbian County, Jianshe Xiang, Aogadaqing, 1500 m a.s.l., 17 June 1953, *P.I. Mao 02129* [young fr.] (IBSC, 0456960 & 0456968; WUK, 0194633, 0205745 & 0246735, images) • Pingbian County, Yaoshan Qu, Chongtou Xiang, Jinzhuliang, 1200 m a.s.l., 17 April 1954, *P.I. Mao 03850* [fr.] (IBSC, 0457693; PE, 02054617; WUK, 0205104, image) • Pingbian County, Yaoshan Qu, Hongtuzhai to Qingjiao, 1300 m a.s.l., 27 October 1992, *Y.M. Shui 000884* [fl.] (PE, 00197738 & 01855940) • Pingbian County, Yuping District [Yuping Town], Pujiqing, 1600 m a.s.l., 29 March 1958, *K.M. Feng 21788* [young fr.] (PE, 00438119) • ibid., 1640 m a.s.l., 12 November 1982, *P.I Mao 82-173* [young fr.] (SWFC, 00004476 & 00004477) • Pingbian County, Yuping Town, Yibaizu Dazhai, Makonglong, Gezu, 22°59′39.3′′N, 103°44′42.1′′E, 1126 m a.s.l., 20 April 2012, *Pingbian Chinese Medicine Resources Exped. 5325230126* [young fr.] (IMDY, IMDY0014685, image) • Ping-bien Hsien [Pingbian County], 1700 m a.s.l., 25 December 1932, *H.T. Tsai 52471* [fr.] (A, 0121925, not seen; IBSC, 0456970; SZ, 0012556) • ibid., 1350 m a.s.l., 20 May 1934, *H.T. Tsai 55337* [young fr.] (A, 00121924, not seen; IBSC, 0456973; LBG, 00080620; PE, 00196993; SWFC; SZ, 00061666) • ibid., 1400 m a.s.l., 3 June 1934, *H.T. Tsai 60089* [young fr.] (A, 0121930, not seen; IBSC, 0456965; LBG, 00080621; SZ, 00061669 & 00125601) • ibid., 1400 m a.s.l., 17 June 1934, *H.T. Tsai 60228* [young fr.] (A, 00121931, not seen; IBSC, 0456972; LBG, 00080638; SZ, 00061664 & 00125646) • ibid., 1400 m a.s.l., 2 July 1934, *H.T. Tsai 60598* [young fr.] (A, 0121927, not seen; IBSC, 0456974; SWFC; SZ, 00125691) • ibid., 1400 m a.s.l., 7 August 1934, *H.T. Tsai 61424* [young fr.] (A, 0121928, not seen; IBSC, 0456971; SZ, 00061667 & 00125736) • ibid., 1300 m a.s.l., 13 August 1934, *H.T. Tsai 61476* [young fl.] (A, 0121929, not seen; IBSC, 0456975; SZ, 00061668 & 00125826) • Hekou County, Kuzhuzhai, 1480 m a.s.l., 6 August 1959, *W.X. Liu 509* [fr.] (HITBC, 031779; IBSC, 0456969; PE, 00438120; WUK, 0277612, image) • Hekou County, Lianhuatan Xiang, Yingpanshan, 1250 m a.s.l., 25 August 1993, *Y.M. Shui 003618* [young fr.] (PE, 02055208 & 02054615) • ibid., 1950 m a.s.l., 10 September 1999, *Y.M. Shui et al. 10782* [young fr.] (PE, 02055209) • Ma-kwan Hsien [Maguan County], 1600 m a.s.l., 2 March 1933, *H.T. Tsai 51888* [young fr.] (A, 0121923, not seen; IBSC, 0456976; PE, 00438121; SZ, 00061665; WUK, 0038093, image) • Maguan County, Gulinqing, nearby Jinchang River, 22°52′1′′N, 104°1′06′′E, 17 October 2022, *Y.M. Shui, W.H. Chen & J.S. Sheng 31744* [young fr.] (PE, 02054618) • Wenshan County, Laojunshan Forest Farm, Yaodian, Dawantian, 1600 m a.s.l., 6 May 1993, *Y.M. Shui 002577* [young fr.] (PE, 02054611) • Xichou County, Fadou Xiang, Caoguoshan, 23°22′13′′N, 104°46′59′′E, 1539 m a.s.l., 9 December 2010, *A.Q. Dong & P.W. Hu F2-008* [st.] (IBSC) • Malipo County, Huangjinyin, 1200 m a.s.l., 13 January 1940, *C.W. Wang 83154* [young fr.] (IBSC, 0457810; SZ, 00125871; WUK, 0269823, image) • ibid., 1100 m a.s.l., 21 January 1940, *C.W. Wang 86320* [fr.] (IBSC, 0457686; SZ, 00135961; WUK, 0275213, image) • ibid., 1400–1800 m a.s.l., 8 November 1947, *K.M. Feng 13066* [fr.] (A, 0121934, not seen; KUN, 0234163; SZ, 00125916) • Malipo County, Laojunshan, Sez-tai-po, 1300–1400 m a.s.l., 20 December 1947, *K.M. Feng 14043* [fr.] (A, 0121933, not seen; SZ, 00126080; WUK, 0194914, image) • ibid., 26 May 1962, *K.M. Feng 22820* [fr.] (PE, 0457836) • Malipo County, Mengdong Xiang, Yezhutang, 8 January 2010, *SE Yunnan Expedition GBOWS538* [young fr.] (KUN, 1279312 & 1279313) • Malipo County, Xiajinchang, Huoshaoliangzi, 19 August 2012, *Y.H. Tong & L. Bai 12081945* [st.] (IBSC). **Vietnam. Lai Chau Province** • Phong Tho, 1500 m a.s.l., 1 December 1937, *E. Poilane 26658* [young fr.] (L, L.3778616, image, L.3778617, not seen; P, P04485543, P04485545, P04485546, P05195569 & P05195570, images) • Than Uyen District, municipality Ho Mit, 22°06′N, 103°52′E, 1400–1500 m a.s.l., 21–22 May 1999, *N.T. Hiep, P.H. Hoang, L.V. Averyanov NTH2665* [fr.] (LE, LE01041978). **Lao Cai Province** • [Bat Xat District], between Trinh Thuong and Muong Hum, 2000 m a.s.l., 9 January 1931, *E. Poilane 18813* [young fr.] (P, P04485542, P04485544, P04485547, P05244318 & P05244319, images) • Bat Xat District, Bat Xat Nature Reserve, 5 km SSE of Y Ty Village, 22°37′17′′N, 103°38′04′′E, 1750 m a.s.l., 19 October 2018, *M.S. Nuraliev 2359* [fl.] (BRIT, BRIT1150599; IBSC; MW, MW0757560) • Bat Xat District, Y Ty commune, Bat Xat Nature Reserve, 22°37′17.3′′N, 103°38′03.7′′E, 1741 m a.s.l., 8 May 2019, *B.H. Quang, T.D. Binh VN-RU 124* [young fr.] (BRIT, BRIT1135765; MW, MW1048025) • ibid., 22°36′17.1′′N, 103°38′44.8′′E, 1445 m a.s.l., 30 July 2019, *B.H. Quang, T.D. Binh VN-RU 175* [young fr.] (BRIT, BRIT1135757; MW, MW1048026) • Bat Xat District, Sang Ma Sao municipality, Bat Xat Nature Reserve, trail to Ky Quan San Mountain, 22°31′15′′N, 103°38′51′′E, 1360 m a.s.l., 4 November 2022, *M.S. Nuraliev, D.F. Lyskov NUR 3724* [fl.] (IBSC; MW, MW1047750); Chapa [Sapa], 1500 m a.s.l., April 1925, *P.A. Pételot 1772* [young fr.] (holotype: UC, UC259692, image; isotypes • P, P00647843, image; US, 00116935, image) • Chapa [Sapa], 1500 m a.s.l., August 1927, *P.A. Pételot 3115* [fl.] (P, P04485549 & P04485550, images) • Chapa [Sapa], 1300 m a.s.l., July 1931, *P.A. Pételot s.n.* [fl. & young fr.] (L, L.2619345, image; NY, 04367530, image; P, P04485548, image; US, 02992181, image) • Sapa to O Qui Ho, 1630 m a.s.l., 8 December 1964, *Sino-Vietnam Exped. 143* [young fr.] (IBSC, 0457761; PE, 02054619) • Sapa, Ham Rong Resort, 22°20′3′′N, 103°50′55′′E, 1650 m a.s.l., 1 May 2014, *Y.H. Tong & D.H. Voung VN025* [young fr.] (IBSC) • Sapa District, Silver Waterfall, 22°21′46′′N, 103°46′37′′E, 1900 m a.s.l., 1 May 2014, *Y.H. Tong & D.H. Voung VN018* [young fr.] (IBSC).

***Vaccinium pseudobullatum*. China. Yunnan Province** • Funing County, Long-May, 1000 m a.s.l., 8 May 1940, *C.W. Wang 87115* [fr.] (paratypes: HITBC, 031712; IBSC, 0457839; KUN, 1208911 & 1208912; SZ 00126428, not seen; WUK, 0276519, image) • Xichou County, Fadou, 23°24′N, 104°46′E, 1250 m a.s.l., 12 December 1939, *C.W. Wang 83119* [st.] (HITBC, 031612; IBSC, 0457793; WUK 0271670, image) • ibid., 26 April 1959, *Q.A. Wu 7724* [fl.] (WUK, 273471, image) • ibid., 23°24′39.5′′N, 104°49′31.8′′E, 1480 m a.s.l., 23 March 2007, *L.M. Gao GLM-07333* [fl.] (KUN, 1272574) • Xichou County, Ma-chia, 14 October 1947, *K.M. Feng 12474* [st.] (PE, 00199938; WUK, 0202926, image) • Xichou County, Xiaoqiaogou, 1700 m a.s.l., 6 April 1959, *Q.A. Wu 7342* [fl.] (WUK, 273384, image) • Malipo County, Huangjinyin, 1100 m a.s.l., 20 January 1940, *C.W. Wang 86300* [st.] (paratypes: IBK, IBK00302353; IBSC, 0002589; KUN, 1208913 & 1208914; SZ 00126470, not seen; WUK, 0275206, image) • Malipo County, Tiechang Xiang, Guan’gao, 1000 m a.s.l., 14 February 1940, *C.W. Wang 86808* [fl.] (HITBC, 031611; IBSC, 0457844; PE, 02054603; WUK, 0275498, image); Malipo County, Tiechang, 1200 m a.s.l., 20 February 1940, *C.W. Wang 86988* [fl.] (holotype: PE, not seen; isotypes: IBK, IBK00302352; IBSC, 0457816; KUN, 12088909 & 12088910; SZ, 00126560; WUK, 0268189, image) • Malipo County, Tung-ting, 1300–1500 m a.s.l., 21 November 1947, *K.M. Feng 13490* [fl.] (paratypes: A, 00121920, not seen; KUN, 1208915 & 1208916; SZ 00126515, not seen) • Malipo County, Xiajinchang Xiang, Huoshaoliangzi, 1400 m a.s.l., 6 July 2009, *Yunnan Expedition YN-ET 871* [fr.] (PE, 02054602) • Malipo County, Xiajinchang Xiang, Yunling Cun to Zhongzhai Cun, 18 August 2012, *Y.H. Tong & L. Bai 12081805* [st.] (IBSC) • Malipo County, Xiajinchang Xiang, Road XH45, No. 33 road pile, 23°9′51.62′′N, 104°50′18.36′′E, 1600 m a.s.l., 18 March 2014, *B. Yang & C.Q. Liu YangbLiu-125* [fl.] (KUN,1249644) • Malipo County, Zhongzhai to Ganbazi, 1940 m a.s.l., 22 March 2002, *Y.M. Shui, W.H. Chen, J.S. Sheng, S.D. Chang & C.L. Fan 20162* [fl.] (KUN, 1276106) • Malipo County [without precise locality], 23°7′3′′N, 104°50′11′′E, 1762 m a.s.l., 7 January 2010, *SE Yunnan Expedition GBOWS132* [young fr.] (PE, 02054604 & 02054604). **Guangxi Zhuang Autonomous Region** • Napo County, Baidu Xiang, Nonghua Cun, Nongbu Tun, 20 May 1977, *Z.G. Wang 3-50862* [fr.] (GXMI, GXMI029232) • ibid., 12 October 1977, *D. Fang 3-1362* [fr.] (GXMI, GXMI029229 & GXMI029230) • ibid., 7 April 1978, *Z.G. Wang 3-1674* [fl.] (GXMI, GXMI029231) • ibid., 1200 m a.s.l., 13 March 1986, *D. Fang & D. H. Qin 79683* [fl.] (GXMI, GXMI029226 & GXMI029227) • ibid., 1200 m a.s.l., 22 May 1989, *South China Expedition 917* [fr.] (IBSC, 0420588) • ibid., 1200 m a.s.l., 28 March 1990, *D. Fang & X.M. Zhong 79707* [fl.] (GXMI, GXMI029228) • ibid., 1200 m a.s.l., 28 March 1990, *Z.F. Wu 90003* [fl.] (GXMG, GXMG0082624) • ibid., 20 October 1990, *D. Fang, L. Zeng & L.S. Zhou 0721* [fr.] (GXMI, GXMI029233) • ibid., 1200 m a.s.l., 28 June 2008, *Guangxi Expedition 1640* [fr.] (IBK, IBK00279961) • ibid., 1200 m a.s.l., 1 March 2017, *B.M. Wang TYH-965A* [fl.] (IBSC) • Napo County, Baidu Xiang, Nonglong Cun, 1250 m a.s.l., 20 August 2009, *W.B. Xu & D.X. Nong 091230* [fr.] (IBK, IBK00279962) • ibid., 23°14′32.99′′N, 105°33′1.68′′E, 1337 m a.s.l., 4 July 2012, *Y.H. Tong & L. Bai 12070410* [fr.] (IBSC) • Napo County, Baihe Gongshe, Shanglong Dadui, 22 April 1977, *Y. Lin 3-5334* [fl.] (GXMI, GXMI027300) • Napo County, Longhe Town, 860 m a.s.l., 23 March 2012, *Y.S. Huang & Y.B. Liao Y1030* [fl.] (IBK, IBK00279959). **Vietnam. Ha Giang Province** • Quan Ba District, Tung Vai commune, Thang village, 23°3′4.2′′N, 104°51′9.7′′E, ca. 1000 m a.s.l., 24 April 2018, *L.V. Averyanov, K.S. Nguyen, T.H. Nguyen, Q.H. Nguyen, Q.N. Chuong & T.V. Maisak VR807* [fl.] (LE, LE01049715) • Quan Ba District, Bat Dai Son, 28°8'11′′N, 105°0'48′′E, 1310 m a.s.l., 6 April 2000, *D.K. Harder, N.T. Hiep, L.V. Averyanov & N.Q. Hieu 5221* [young fr.] (P, 06636453) • Quan Ba District, Bat Dai Son Nature Reserve, San Chu Village, 23°8'55.1′′N, 104°59'45.7′′E, 1100–1290 m a.s.l., 13 April 2018, *L.V. Averyanov, K.S. Nguyen, T.H. Nguyen, Q.N. Chuong & T.V. Maisak VR021* [fl.] (HN; LE, LE01054025, LE01054026 & LE01067170) • Quan Ba District, Bat Dai Son Nature Reserve, Thanh Van commune, Mo Sai village, 23°7′46.4′′N, 104°57′56.2′′E, ca. 1000 m a.s.l., 18 April 2018, *L.V. Averyanov, K.S. Nguyen, T.V. Maisak & Q.N. Chuong VR387* [fl.] (HN; LE, LE01067273, photos LE01061504; MW, MW1045975) • Bac Me District, Phieng Luong commune, Phieng Luong village (border with Tuyen Quang Province, Na Hang District, Khuoi Phin), 22°37′48.3′′N, 105°19′32.4′′E, 1183 m a.s.l., 7 April 2014, *N.Q. Hieu, N.T. Hiep, N.N. Hung, T.Q. Ngan, T.P. Tuan, G.M. Pao CPC 7301* [fr.] (LE, LE01167473). **Cao Bang Province** • Bao Lac District, Hong An municipality, Mi Lung village, 22°50′1.3′′N, 105°50′5.7′′E, ca. 1000 m a.s.l., 20 November 2014, *L.V. Averyanov, N.T. Hiep, N.S. Khang, T. Maisak, L. Osinovetz CPC7515* [fr.] (LE, LE01167480; P, P00978630) • Bao Lac [Nguyen Binh] District, Yen Lac municipality, vicinities of Yen Lac village, 22°44′N, 105°50′E, ca. 1000 m a.s.l., 15 November 1998, *L.V. Averyanov, N.T. Hiep, P.K. Loc, N.X. Tam CBL266* [st.] (LE, LE01041984) • Nguyen Binh District, Ca Thanh municipality, 22°44′N, 105°50′E, ca. 1000 m a.s.l., 13 April 1999, *P.K. Loc, P.H. Hoang, L.V. Averyanov CBL1287* [fl.] (LE, LE01041967; MW, MW1045966). **Bac Kan Province** • Cho Don District, Ban Thi Municipality, Phia Khao village, 22°17′31′′N, 105°31′51′′E, 800–900 m a.s.l., 24 May 2004, *L. Averyanov, N.T. Hiep, P.V. The, N.T. Vinh HAL 4862* [fr.] (LE, LE01167476 & LE01167477).
